# Environmental DNA from Seawater Samples Correlate with Trawl Catches of Subarctic, Deepwater Fishes

**DOI:** 10.1371/journal.pone.0165252

**Published:** 2016-11-16

**Authors:** Philip Francis Thomsen, Peter Rask Møller, Eva Egelyng Sigsgaard, Steen Wilhelm Knudsen, Ole Ankjær Jørgensen, Eske Willerslev

**Affiliations:** 1 Centre for GeoGenetics, Natural History Museum of Denmark, University of Copenhagen, Copenhagen, Denmark; 2 Section for EvoGenomics, Natural History Museum of Denmark, University of Copenhagen, Copenhagen, Denmark; 3 Technical University of Denmark, National Institute of Aquatic Resources, Charlottenlund, Denmark; 4 Greenland Institute of Natural Resources, Nuuk, Greenland; 5 Department of Zoology, University of Cambridge, Cambridge, United Kingdom; 6 Wellcome Trust Sanger Institute, Wellcome Genome Campus, Hinxton, Cambridge, United Kingdom; Central Michigan University, UNITED STATES

## Abstract

Remote polar and deepwater fish faunas are under pressure from ongoing climate change and increasing fishing effort. However, these fish communities are difficult to monitor for logistic and financial reasons. Currently, monitoring of marine fishes largely relies on invasive techniques such as bottom trawling, and on official reporting of global catches, which can be unreliable. Thus, there is need for alternative and non-invasive techniques for qualitative and quantitative oceanic fish surveys. Here we report environmental DNA (eDNA) metabarcoding of seawater samples from continental slope depths in Southwest Greenland. We collected seawater samples at depths of 188–918 m and compared seawater eDNA to catch data from trawling. We used Illumina sequencing of PCR products to demonstrate that eDNA reads show equivalence to fishing catch data obtained from trawling. Twenty-six families were found with both trawling and eDNA, while three families were found only with eDNA and two families were found only with trawling. Key commercial fish species for Greenland were the most abundant species in both eDNA reads and biomass catch, and interpolation of eDNA abundances between sampling sites showed good correspondence with catch sizes. Environmental DNA sequence reads from the fish assemblages correlated with biomass and abundance data obtained from trawling. Interestingly, the Greenland shark (*Somniosus microcephalus*) showed high abundance of eDNA reads despite only a single specimen being caught, demonstrating the relevance of the eDNA approach for large species that can probably avoid bottom trawls in most cases. Quantitative detection of marine fish using eDNA remains to be tested further to ascertain whether this technique is able to yield credible results for routine application in fisheries. Nevertheless, our study demonstrates that eDNA reads can be used as a qualitative and quantitative proxy for marine fish assemblages in deepwater oceanic habitats. This relates directly to applied fisheries as well as to monitoring effects of ongoing climate change on marine biodiversity—especially in polar ecosystems.

## Introduction

Among the effects of climate change is an increased northward dispersal of a large number of marine taxa [1,2]. While a few commercial fish species are relatively well monitored by national (e.g. GINR—Greenland Institute of Natural Resources) and international agencies (e.g. ICES—International Council for the Exploration of the Sea, and NAFO—Northwest Atlantic Fisheries Organization), a large number of taxa and huge areas of ocean are almost never studied. This is mainly due to a lack of economic interests, great water depths and substrates unsuitable for bottom trawling, such as steep slopes and deepwater coral cover in Greenland [3]. Existing distribution data can be combined with climate data and models to predict the future development of fish faunas, including the expansion of many boreal species into the Arctic Ocean [4]. Such forecasts are in high demand among managers, decision makers and politicians, but the models are easily hampered by lack of input data [5]. Lack of input data for marine fishes is mainly due to i) lack of access to existing data [6], ii) lack of taxonomic expertise on fisheries surveys [7] and most importantly iii) lack of surveys. As pointed out recently, novel survey approaches are necessary to follow the fauna development in remote areas [8]. Unfortunately, such surveys are extremely expensive and only a few countries are currently able to contribute. Especially deepwater habitats are expensive to survey due to the long setting and hauling time of the trawls.

Fish and shrimp stocks on the Greenland continental shelf and slope have been monitored by annual bottom trawl surveys since 1988 [9], and the fish fauna is probably among the more well-known deepwater fish faunas globally. More than 270 fish species have been recorded [10], and new species are reported almost annually, due to climate change and as a result of taxonomic revisions (e.g. [11]). The main targets of both scientific surveys and commercial fisheries are Greenland halibut (*Reinhardtius hippoglossoides*), rockfishes (*Sebastes* spp.), Atlantic cod (*Gadus morhua*) and deepwater prawn (*Pandalus borealis*), but a number of non-commercial deep-sea species are also caught in high quantities in the trawls [12,13]. Both shelf and slope species are considered in serious decline due to commercial fishing and perhaps also climate change both in Greenland [9,14] and in nearby Canadian waters [15]. Some species have not declined [9], perhaps because the part of the Davis Strait that belongs to Greenland is characterized by rocky bottoms unsuitable for bottom trawling and because many of the species are recruited from outside the commercial fishing areas and brought to the Davis Strait by currents. While areas with rough bottoms might act as natural Marine Protected Areas, they are also almost impossible for fisheries biologists to study and monitor with traditional methods. Thus, alternative efficient and non-invasive techniques for qualitative and quantitative oceanic fish surveys are needed [16].

A new alternative or supplementary method for monitoring fish communities is sequencing of environmental DNA (eDNA) from water samples [17–20]. The main advantages of this approach are i) higher efficiency compared to traditional methods [18,19,21], ii) non-invasive nature of the methodology, and iii) ease of standardization across study areas and sampling personnel. However, only a few studies so far have focused on marine eDNA from macroorganisms [19,22–26], and both the quantitative aspect of the eDNA, and deep water (>50 m) environments have remained almost unexplored. This is despite one of the most obvious applied perspectives of aquatic eDNA, which lies in the potential assessment of marine fish stocks [19,20,27]. In freshwater systems, several studies suggest that eDNA can be used quantitatively, but for relative rather that absolute quantification [18,28–30]. The degradation of eDNA in aquatic systems has been found to occur at a scale of days or weeks [18,19,31,32], rendering long-distance dispersal unlikely. This is particularly important in large open systems such as the oceans, where sea currents could potentially transport eDNA over large distances.

Here we report eDNA metabarcoding of seawater samples from continental slope depths (188 to 918 m) in the Davis Strait off Southwest Greenland and compare eDNA sequence reads to parallel catch data from trawling. We discuss the results in light of the perspectives and limitations of using eDNA sequence read abundance as a proxy for marine fish abundance.

## Materials and Methods

### Trawling survey

**Fig 1:**
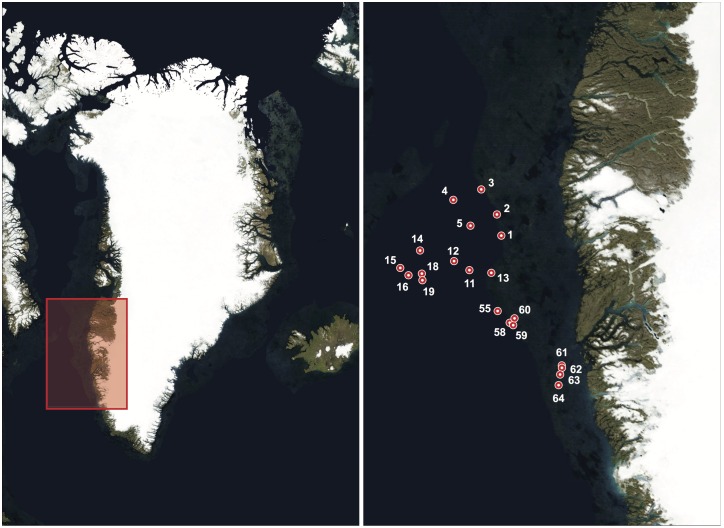
Map of sampling sites. Overview of study site in Greenland (left), and detailed map of sampling sites in the Davis Strait, SW Greenland (right). Numbers corresponds to the sampling sites as in Table A in S1 File. Map: NASA, Visible Earth, Blue Marble.

Trawling was performed from R/V Paamiut in 2012, as a part of annual bottom-trawl surveys off SW Greenland (NAFO division 1C-1D (62830′ N–66815′ N [0]) [33] conducted by GINR (Fig 1, Table A in S1 File), which has permission to carry out surveys in all Greenland waters with small mesh gear. The trawl had 140-mm meshes, with a 30-mm liner in the codend, rockhopper ground gear and a wingspread of 20–25 m. Towing time was 30 minutes but towing times down to 15 minutes were accepted. Towing speed was about 3 knots. Abundance and biomass estimates were standardized to catch per km^2^ swept area using exact towed distance and wingspread. For further information about trawl gear, see [33]. The catchability of all species was assumed to be 1.

### Water sampling

Samples were collected using a Nansen metal water sampler. Twenty-one seawater samples, each of 2 L, were collected immediately before each bottom trawl at the same position as the trawling (Fig 1). The water sampler was cleaned with freshwater between each trawl, but due to pressure at high depths, the sampler had to stay open on the way down to the chosen collection depth. This may have resulted in small amounts of water from just above sampling depth being mixed with the water sampled from the chosen depth. However, we do not currently have a better solution for sampling deep water. Samples were immediately transferred to 2 L sterile plastic containers, closed and stored at -20°C until filtering in the laboratory. Samples were collected by OAJ using single-use nitrile gloves.

### Water filtration and eDNA extraction

Filtration and DNA extractions were performed in the laboratories at the Centre for GeoGenetics, which are dedicated for working with low concentration samples. Regular decontamination routines are in place, including UV-light, and pre- and post-PCR work is separated. Samples were thawed at room temperature and shaken thoroughly to increase homogenization before filtering. For each sample, 1.5 L of seawater were vacuum-filtered onto 47 mm diameter 0.45-μm pore size nylon filters (Osmonics, Penang, Malaysia), and DNA was extracted using bead beating and the Qiagen DNeasy^®^ Blood & Tissue Kit as described in [19], with final elution steps of 2x60 μL AE Buffer for each sample. The filter funnel and associated parts as well as tweezers and scissors were cleaned using tap water, 10% bleach and 70% ethanol after each filtration. Tweezers and scissors were moreover de-contaminated using 96% ethanol and burning. Three filtration and extraction blanks consisting of clean, deionized water samples stored together with the field samples were filtered and extracted as the first, middle and last sample.

### PCR amplification

For the eDNA metabarcoding approach, we used tagged versions of a recently published fish-specific primer set targeting a small region of the mitochondrial DNA 12S rRNA gene [21]. The primers (teleo_F: 5′-ACACCGCCCGTCACTCT and teleo_R: 5′-CTTCCGGTACACTTACCATG), amplify an approximately 100 bp product, and are used together with a human blocking primer (teleo_blk: 5′-ACCCTCCTCAAGTATACTTCAAAGGAC-SPC3I). This primer set was developed for metabarcoding and has proven efficient for the approach. Tags were designed using the OligoTag program [34], and consisted of six nucleotides with a distance of at least three bases between any two tags. Tags were preceded by two or three random bases; NNN or NN [35], and identical tags were used on the forward and reverse primers for a given sample. PCR reactions were carried out in four replicates per sample, using the same tag for PCR replicates, but a unique tag for each sample. PCR reactions were performed in 25 μL volumes of 3 μL template DNA extract, 10 μL TaqMan Environmental MasterMix 2.0 (Life Technologies), 10 μL ddH_2_O, 0.5 μL of each primer (10 μM), 0.5 μL human blocking primer (100 μM) and 0.5 μL HL-dsDNase (ArcticZymes) (5U/μL). Before DNA extract was added, the reactions were stored at 37°C for 15 minutes and 60°C for 15 minutes for activation and inactivation of the DNase, respectively. The DNase removes any double-stranded DNA (potential contamination) from the reactions before the target DNA template is added. Thermocycling parameters were: 95°C for 10 minutes, 40 cycles of 94°C for 30 s, 55°C for 30 s, 72°C for 1 minute, and a final elongation of 72°C for 5 minutes. PCR products were mixed in four pools each containing one PCR replicate of each sample (6 μL per replicate), such that the same tag was added only once to each pool. The pools were then purified using Qiagen’s MinElute PCR purification kit.

A mock sample (positive control) was prepared using tissue-derived DNA from five species of common Northern European freshwater fish—all absent in the Davis Strait. We used fish species with distributions and habitats different from the target species, in order to avoid false positives in the water sample data. Since the primers used here are generic fish primers, differences in primer affinity between mock species and target species should not be an issue. The mock was made so that, based on Qubit fluorometer (Invitrogen) measurements, final concentrations of DNA ranged between 0.2 and 5.5 ng/uL (*Silurus glanis* 0.2 ng/uL, *Abramis brama*: 1.4 ng/uL, *Gobio gobio*: 2.7 ng/uL, *Tinca tinca*: 4.8 ng/uL, and *Gymnocephalus cernua* 5.5 ng/uL, and). The mock sample was amplified as described above and sequenced along with the water samples using a unique tag. The four PCR replicates of the mock were distributed across the four pools of PCR replicates from the water samples. Tissue from *Lota lota* was included in the mock, but sequences from this species were removed from the mock results, since the primers were found to have mismatches to *Lota lota*, most likely resulting in unequal amplification. Nevertheless, this species’ DNA as well as the background level of DNA from a fish contaminant (*Oncorhynchus* sp.) also found in the mock sample, may imitate a more natural situation of true and complex eDNA samples, where DNA from several species (target and non-target) is present.

Two negative PCR controls (blanks) were included for each round of PCR amplification, using unique tags, and added to the pools of the eDNA PCR replicates.

Fragment sizes were verified on 2% agarose gels stained with GelRed^™^.

### Library preparation and high-throughput sequencing

Library building was performed on the purified pools of PCR products using the TruSeq DNA PCR-free LT Sample Prep kit (Illumina). A total of four libraries (corresponding to the four pools) were constructed. Each library thus included one replicate of every sample, two PCR blanks, and one mock sample replicate. Additionally, each library contained one replicate of each of eight samples from another research project. The manufacturer’s protocol was followed with the exception that samples were incubated with the elution buffer over two rounds of 37°C for 10 minutes. Approximately 250 ng of PCR product from each pool was used as input for the libraries, and a library blank was included. The concentration and fragment size distribution of the libraries were verified on an Agilent 2100 Bioanalyzer. Libraries were pooled in equimolar concentrations and sequenced on the Illumina MiSeq platform (½flow cell) at the Danish National Sequencing Centre, applying 150 bp Paired-End sequencing. A spike-in of PhiX was used to increase complexity in the runs.

### Reference database

A number of species caught in the trawls as well as some species common to Greenland were not represented in public databases (European Molecular Biology Laboratory (EMBL), National Center for Biotechnology Information (NCBI)) for the targeted 12S rRNA gene region. Tissue samples of 45 fish species from the Natural History Museum of Denmark (NHMD) fish collection were used to obtain the remaining 12S DNA sequences (Table B in S1 File). Samples had been stored in 96% EtOH at -20°C. DNA was extracted from muscular tissue using Qiagen’s DNeasy^®^ Blood & Tissue kit, following the manufacturer’s instructions, in a room dedicated to DNA extraction.

PCR reactions targeting a ca. 600 bp product of the 12S rRNA gene were performed using primers teleo_R and V05F_898 (5’-AAACTCGTGCCAGCCACC). PCRs were performed in 25 μL total reactions of 1 μL template DNA extract, 10 μL TaqMan Environmental MasterMix 2.0, 13 μL ddH_2_O and 0.5 μL of each primer (10 μM). Thermocycling parameters were: 95°C for 10 minutes, 45 cycles of 94°C for 30 s, 54°C for 30 s, 72°C for 1 minute, and a final elongation step of 72°C for 5 minutes. Fragment sizes were verified on 2% agarose gel stained with GelRed^™^. The PCR products were purified and Sanger sequenced commercially by Macrogen Europe using primers V05F_898 and V05F (5’-CTAGAGGAGCCTGTTCTA). Sequences were trimmed and quality checked using Geneious v. 7.1.7 (Biomatters Ltd.) and authenticated by blast algorithm search against the NCBI nucleotide database (Table B in S1 File). Full sequences were uploaded to Genbank (NCBI Accession no. KX929879-KX929923).

### High-throughput sequencing data analyses

Illumina sequences were analyzed using OBITools [36]. Paired-end reads were initially assembled using the “illuminapairedend” command with a threshold score of minimum 40. Tags and primer sequences were removed from the reads using “ngsfilter”, reads were compiled using “obiuniq” and only sequences with a count of at least 10 and a length of 40–300 bp were retained. Finally, reads were cleaned for variants with a threshold of r = 0.05 using “obiclean”. Final reads were taxonomically assigned by an “ecotag” search against a local ecoPCR database containing reference 12S rRNA sequences of vertebrates, extracted from the EMBL database version r128 (June 2016), as well as 45 sequences of Greenlandic marine fishes produced for this study (Table B in S1 File), and 22 sequences of common Danish marine fishes [21].

To remove errors generated during PCR and sequencing [37–39] from the data, we identified, in the mock sample of each library, the most abundant sequence that had <100% identity to a known species added to the mock sample. We then compared the read count of this sequence to the total read count from the mock sample. We thereby obtained a putative error rate of between 0.028% - 0.128% across the four libraries. This rate was used to clean the data, such that sequences occurring at a lower count than the calculated library-specific error threshold were removed from the data. After cleaning, reads were initially categorized as: i) authentic reads, ii) obvious contaminants and spurious reads, and iii) reads from the mock. This assignment was done in accordance with the recorded occurrences of marine fishes in the seas around Greenland [10,12] (Table C in S1 File), as well as consideration of common contaminants in DNA studies [40] (see Results section). All reads were assigned to taxon based on 90–100% sequence similarity. Some of the assignments based on eDNA were later reassigned to a higher or lower taxonomic level based on reference database coverage for the taxon in question and on knowledge of Greenland’s fish fauna composition and the commonness of different species (Table 1, Table C in S1 File).

**Table 1:** Overview of results. Table shows the fish families detected by trawling and eDNA, respectively, as well as lower taxonomic assignments within each family. For each taxon, the total catch number of individuals and biomass for trawling, and total number of eDNA reads for water samples is given. Some taxa were only found with eDNA (^1^) and some were only found with trawling (^2^). For some eDNA assignments, the initial taxonomic identification was either downgraded to family level (*) or upgraded to genus/species level (§), based on the taxonomic coverage of the reference database and occurrences of Greenlandic fish species (Table C in S1 File). The table also shows the number of samples (out of 21) that a family was detected in using trawling and eDNA, respectively.

Family	Species	Detection		Total catch (family) (per km^2^)		Total eDNA (family)	No. of positive samples (family)	
		Trawl	eDNA	Biomass (kg)	Number of individuals	Number of reads	Trawl	eDNA
Alepocephalidae	*Alepocephalus agassizii*	x	*Alepocephalus* sp.	33.8	141.6	4408	2	1
Ammodytidae^1^	*Ammodytes* sp.^1^ §		x	NA	NA	4668	0	2
Anarchichadidae	*Anarhichas denticulatus*	x	*Anarhichas* sp.	725.5	193.7	23976	8	7
	*Anarhichas minor*	x						
Argentinidae	*Argentina silus*	x	x	10.5	82.8	2394	2	1
Arhynchobatidae	*Bathyraja spinicauda*	x	x	1576.9	83.9	63261	3	7
Bathylagidae	*Bathylagus euryops*	x	x	172.7	4445.2	26670	11	7
Clupeidae^1^	*Clupea harengus*^1^ §		x	NA	NA	3351	0	1
Cyclopteridae	*Cyclopterus lumpus*	x	x	6.3	13.6	20329	1	4
Etmopteridae	*Centroscyllium fabricii*	x	x	1029.5	1116.4	14442	11	8
Gadidae	*Boreogadus saida*	x	Gadidae	1002.3	2085.0	31309	5	15
	*Gadus morhua*	x						
	*Melanogrammus aeglefinus*	x						
	*Micromesistius poutassou*	x						
Gaidropsaridae	*Gaidropsarus argentatus*	x	*Gaidropsarus* sp.	18.2	75.5	25913	2	5
	*Gaidropsarus ensis*	x						
Gonostomatidae	*Cyclothone microdon*	x	x	0.2	59.2	2265	3	4
	*Sigmops bathyphilus*	x	x					
Liparidae	*Paraliparis* sp.	x	Liparidae sp.*	0.1	13.2	24971	1	1
Lotidae	*Molva dypterygia*	x	x	49.1	13.6	8055	1	2
Macrouridae	*Coryphaenoides guentheri*	x	*Coryphaenoides* sp.	3039.6	17760.6	69667	18	11
	*Coryphaenoides rupestris*	x						
	*Macrourus berglax*	x	x					
	*Nezumia bairdii*^2^	x						
	*Trachyrincus murrayi*^2^	x						
Moridae	*Antimora rostrata*	x	x	170.6	933.4	1775	12	1
	*Lepidion eques*^2^	x						
Myctophidae	*Benthosema glaciale*^2^	x		118.7	10143.7	10071	18	7
	*Lampanyctus crocodilus*	x	*Lampanyctus* sp.					
	*Lampanyctus intricarius*	x						
	*Myctophum punctatum*	x	x					
	*Notoscopelus kroyeri*	x	*Notoscopelus* sp.					
Notacanthidae	*Notacanthus chemnitzii*	x	x	112.6	249.7	69289	9	11
	*Polyacanthonotus rissoanus*^1^		x					
Notosudidae	*Scopelosaurus lepidus*	x	x	23.4	200.2	848	6	1
Oneirodidae^1^	Oneirodidae sp.^1^ *		x	NA	NA	77	0	1
Osmeridae	*Mallotus villosus*	x	x	29.1	2793.8	10924	3	6
Paralepididae	*Arctozenus risso*^2^	x	Paralepididae sp.*	2.9	46.4	247967	3	7
	*Magnisudis atlantica*	x						
Platytroctidae^2^	*Maulisia mauli*^2^	x		0.6	12.0	NA	1	0
Pleuronectidae	*Hippoglossoides platessoides*	x	x §	19537.6	24083.4	914256	21	20
	*Hippoglossus hippoglossus*^1^ §		x					
	*Reinhardtius hippoglossoides*	x	x					
Psychrolutidae	*Cottunculus thomsonii*	x	*Cottunculus* sp.*	0.1	12.0	1456	1	1
Rajidae	*Amblyraja radiata*	x	*Amblyraja* sp.	145.1	377.6	2473	6	4
	*Rajella fyllae*^2^	x						
Sebastidae	*Sebastes norvegicus*	x	*Sebastes* spp.	9664.8	28515.6	264308	14	19
	*Sebastes mentella*	x						
Serrivomeridae^2^	*Serrivomer beanii*^2^	x		0.5	12.0	NA	1	0
Somniosidae	*Somniosus microcephalus*	x	x §	14631.3	34.0	197841	1	18
Stomiidae	*Borostomias antarcticus*	x	x	15.4	303.2	1923	6	3
	*Chauliodus sloani*^2^	x						
	*Stomias boa*	x	x					
Synaphobranchidae	*Synaphobranchus kaupii*	x	x	36.7	257.4	4151	6	6

Illumina MiSeq raw sequence data are available from the Dryad Digital Repository (http://dx.doi.org/10.5061/dryad.ch576).

### Statistical analyses

Due to the relatively low species-level resolution of the primers for some families (e.g. species in Gadidae), eDNA reads were analyzed both at the family level as well at the level of lowest possible taxonomic assignment (Table 1). For each family/taxon, final reads were summed over all four libraries. A linear model was used to test for effects of biomass and abundance on eDNA read output, while adjusting for the effect of water sampling depth. Both the dependent and independent variable were log transformed, since initial data deviated from a normal distribution (left skewed) (Figure A in S1 File). All reported R^2^ values were adjusted to the number of predictors (adjusted R^2^). Heat maps were constructed in RStudio V. 0.98.1028. Bathymetric data from the ETOPO1 database hosted on the National Oceanic and Atmospheric Administration (NOAA) website (www.noaa.gov) [41] was mapped using the package “marmap” [42] and map data for land mass was added with the package “mapproj”. Relative eDNA counts were interpolated between sampling sites using the “akima” package, and eDNA and trawl data were then plotted with the function “filled.contour” of the “graphics” package. All statistical analyses were performed in R v. 2.13.1 (R Core Team, 2016), unless otherwise stated.

## Results

### Trawling data

A total of 4,412 individuals representing 49 fish species and 28 families were caught by trawling at water depths ranging from 188–918 m with a mean depth of 671 m (Table 1, Table A in S1 File). The total biomass catch was 2,341 kg.

### Qualitative eDNA results

A total of 7,426,375 raw paired-end reads were produced on the Illumina MiSeq platform, of which approximately 83% belonged to samples from this study. We obtained similar sequencing depth across the four libraries (PCR replicates): 1,856,594 +/- 61,664 reads (mean +/- SEM). After data cleaning, a total of 3,551,270 reads were retained from the four libraries. Each sample had similar sequence depth with 161,421 +/- 21,178 final reads (mean +/- SEM). After cleaning, some contaminant sequences remained in the data from water samples as well as from the mock. The most abundant contaminant was *Homo sapiens*, occurring in all samples except the mock, despite the use of human blocking primer. Chicken (*Gallus gallus*) also occurred in several samples, rock pigeon (*Columba livia*) occurred in three samples and a duck (Anatidae, 100% match to *Anas* spp. and *Tadorna tadorna* in NCBI) occurred in one sample. These are all considered lab contaminants. Two critical sample contaminations were the occurrence of the tropical lionfish *Pterois volitans* (Scorpaenidae) in a single sample and the freshwater fish *Phoxinus* sp. (Cyprinidae) in two samples. These contaminants are most likely due to previous work on these species in the same lab. We also retained a very low number (12 copies) of common carp (*Cyprinus carpio*), which could also stem from previous work in the lab. We occasionally observe contamination from commercial consumable fishes (e.g. Salmonidae) in PCRs targeting fish (unpublished data). From the mock sample, only added fish species were retained post cleaning except for a single fish contaminant (*Oncorhynchus* sp.). The occurrence of this species most likely is a result of contamination of the DNA extract of one of the species added to the mock. Only the mock sample contained reads from the freshwater fish species added to the mock. A single authentic species seems to be lost as a result of the cleaning–*Lepidion eques* (Moridae) (1 sample), which was caught by trawling and could thus be expected in the eDNA. After removing human and chicken contaminants along with spurious fish reads, 2,053,277 final reads and 357,213 final mock reads remained.

**Fig 2:**
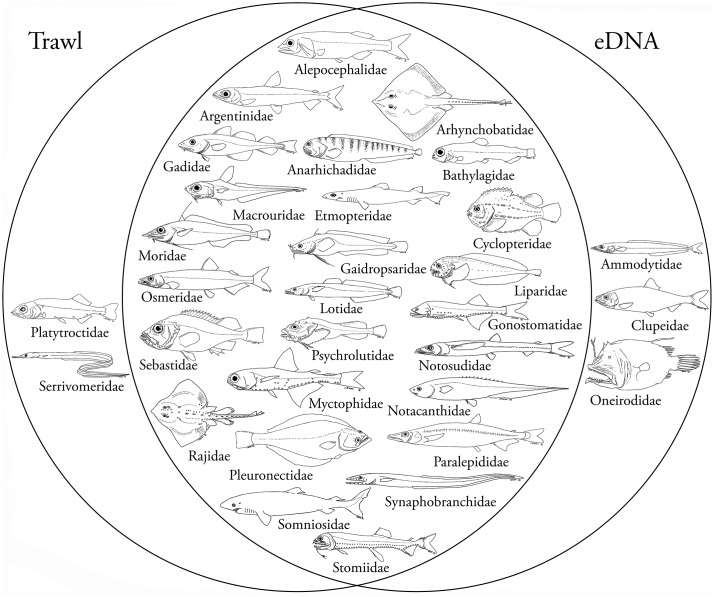
Overview of results from trawling and eDNA. Venn diagrams showing overlap between the qualitative results obtained from eDNA metabarcoding of seawater and trawling, respectively. 26 families were detected using both methods, while three families were only detected using eDNA and two families were only detected using trawling. All drawings by SWK.

**Fig 3:**
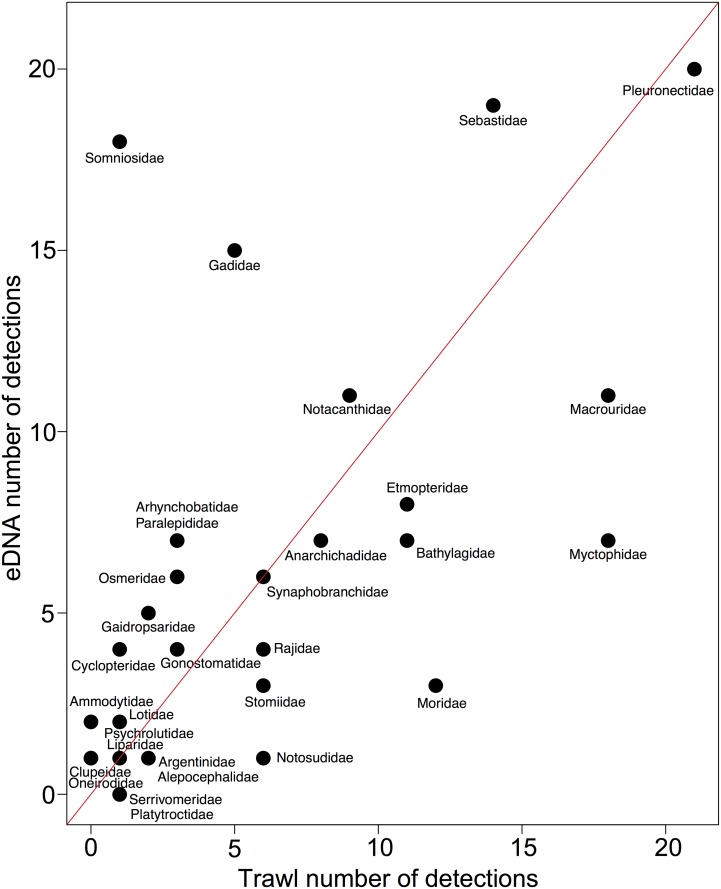
Detections of fish families using eDNA and trawl. Relationship between number of samples positive for eDNA and for trawling, respectively. Maximum number of detections is 21. The red line (y = x) separates the families into those better detected using trawling (below) and those better detected using eDNA (above).

From the final data, eDNA sequences from 37 taxa in 29 Greenlandic fish families were obtained (Table 1). Of these, 26 families were also found with trawling, while 3 families were only found with eDNA (Table 1, Fig 2). There was a positive relationship between the number of samples a family was detected in when using trawling and when using eDNA (p<0.0005, R^2^ = 0.34) (Fig 3). 43% of the final authentic reads belonged to a single species (Greenland halibut) (Table 1). In addition to fish sequences, we found eDNA from thick-billed murre (*Uria lomvia*) in one sample. All PCR and extraction blanks were negative.

### Quantitative eDNA results

**Fig 4:**
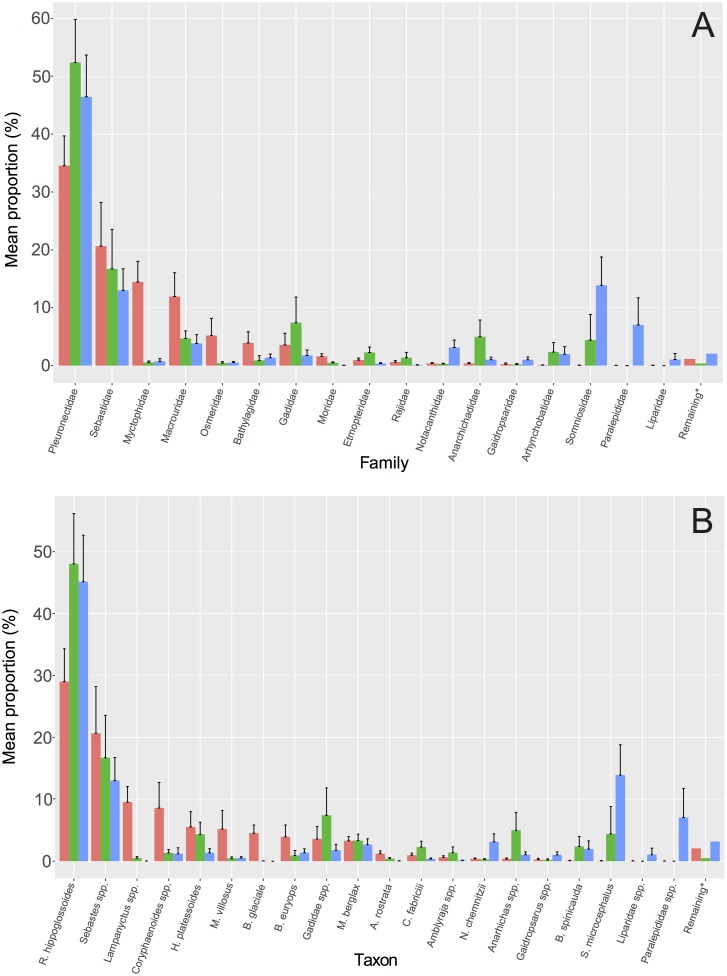
Overview of quantitative results. Barplot of mean + SE of relative fish abundance (red), biomass (green) and eDNA read abundance (blue) across all samples. Data shown for families (A) and lower taxonomic resolution (B). Taxa for which the means of all three variables are <1% are shown together (remaining*) as summed means. Only taxa that are found using both methods are included. Full list of taxa is given in Table 1.

**Fig 5:**
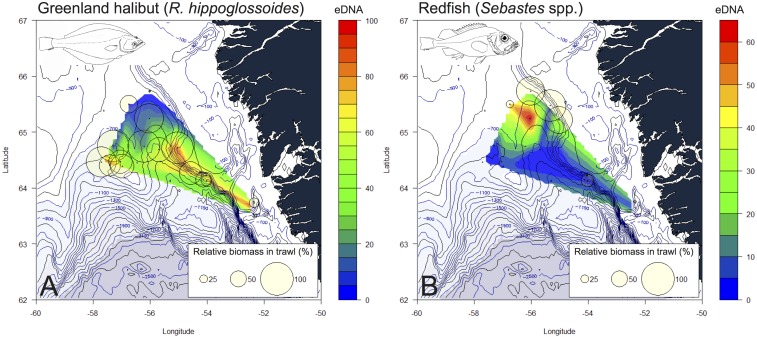
Interpolated eDNA abundances. Heat maps for the two important commercial species, showing interpolated eDNA read abundance (colour) and catch biomass (circles). The size of the circles indicates the relative catch size in % for the species in the given sample, and colour (blue to red) indicates interpolated relative eDNA read abundance. A) Greenland halibut (*Reinhardtius hippoglossoides*), B) redfishes (*Sebastes* spp.). Fish drawings by SWK. Maps: [41,42]

**Fig 6:**
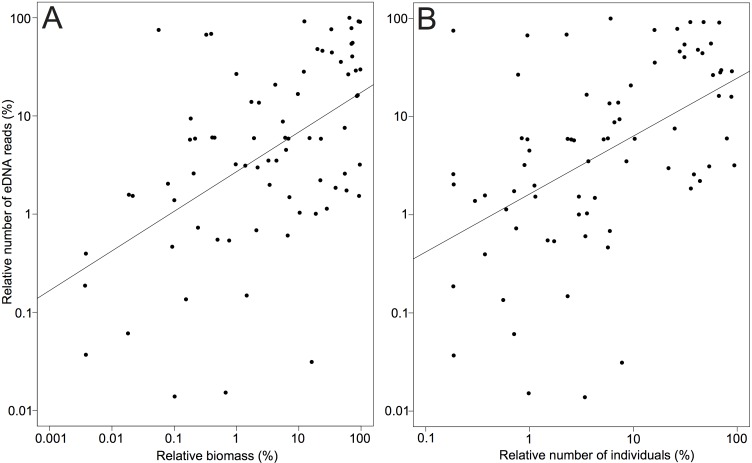
Environmental DNA and fish density. Relationship between relative eDNA read frequencies and relative biomass (A) as well as relative abundance (number of individuals) (B). Results are shown for all fish families detected using both methods across all samples. Regression lines are shown, and a linear model (on log transformed data) showed that eDNA reads correlated with biomass (p<0.0001, R^2^ = 0.26) and number of individuals (p<0.0001, R^2^ = 0.24).

Overall, there was a relatively good correspondence between eDNA read abundance and trawling data (Fig 4). Interpolated eDNA read abundances for Greenland halibut and redfish, the two important commercial species caught in large numbers, showed good accordance with biomass catch data (Fig 5). At the family level, eDNA reads correlated with biomass (p<0.0001, R^2^ = 0.26) and abundance data (p<0.0001, R^2^ = 0.24) obtained from trawling (Fig 6) (for results from individual samples see Figures B-C in S1 File). These results were consistent at a lower taxonomic level (lowest possible of species/genus/family) for both biomass (p<0.005, R^2^ = 0.10) and abundance (p<0.001, R^2^ = 0.14) (Figure D in S1 File). The effects of biomass and number of individuals on eDNA read abundance remained significant after adjusting for the effect of water sampling depth, both at the family level (biomass: p<0.0001, R^2^ = 0.27, individuals: p<0.0001, R^2^ = 0.26) and at lower taxonomic level (biomass: p<0.01, R^2^ = 0.11, individuals: p<0.001, R^2^ = 0.16). In no cases did sampling depth have a significant effect on eDNA abundances (p>0.1). The Greenland halibut was the most abundant species both in terms of individuals, biomass, and eDNA reads (Fig 4, Table 1). The second highest mean catch of individuals and biomass in the trawl was obtained for the rockfishes (*Sebastes* spp.), which were the third most abundant taxon in terms of eDNA reads, closely following the second most abundant species for eDNA, the Greenland shark (*Somniosus microcephalus*) (Fig 4). Only a single individual was caught of the Greenland shark, and the high abundance of the species in the eDNA is thus an exception to the overall pattern found in the study (see Discussion). Read abundance results from the positive mock sample with DNA from known fish species in different quantities, correlated with calculated initial DNA concentrations (p<0.05, R^2^ = 0.81) (Figure E in S1 File).

## Discussion

Marine fishes remain one of the most debated groups of organisms, not least due to the tremendous importance of fisheries for global economy and human health [43]. Nonetheless, fish species and populations worldwide are declining due to over-exploitation, destruction of marine habitats and pollution of the seas [44–47].

The present study demonstrates that sampling of eDNA using basic water samplers can be a useful supplement to scientific deepwater trawling and commercial deep-sea fish stock assessments. Such a non-invasive approach could become highly useful for better estimation of distribution and stocks of commercial as well as non-commercial fishes.

### Trawl vs. eDNA

The two sampling methods showed good overlap altogether (Fig 2). Out of the 28 fish families caught by trawling, 93% were also found with eDNA. The fact that the two families not recovered with eDNA—Platytroctidae and Serrivomeridae—were among the lowest catches in terms of total biomass and were each caught as one individual in a single sample, suggests that eDNA from these families was likely rare or simply non-existent in the water samples (Table 1). *Maulisia mauli* (Platytroctidae) is rare in the area, whereas *Serrivomer beanii* (Serrivomeridae) is a more common, but primarily pelagic species [10]. Pelagic species are generally expected to be very rare in the trawl, which mainly catches fish near the bottom. The three taxa/families detected with eDNA, but not caught in trawls, are either mostly pelagic such as the Atlantic herring (*Clupea harengus*—Clupeidae) and/or primarily occur on shallow banks, like Ammodytidae, or at very low densities as is the case for Oneirodidae [10]. Some of the most apparent incongruences between eDNA read abundance and biomass was seen for Greenland shark and Barracudinas (Paralepididae) (Figs 3 and 4). The Greenland shark was detected in several eDNA samples despite only being caught as a single individual with trawling. This species, which probably easily avoids trawls, still leaves significant traces of eDNA (see below). Barracudinas are common in the area, but are pelagic species, which likely explains the abundance of the family in eDNA but not in trawling.

The eDNA obtained from thick-billed murre in one sample, demonstrates the versatility of the approach to obtain information from other taxa than the ones primarily under study [18,19]. The thick-billed murre is a common breeding bird in Greenland.

It has been demonstrated that detection of rare taxa with the eDNA approach is greatly improved by filtering larger water volumes (up to 100 L) and performing more PCR replicates [21], than applied here. We expect that a larger water volume and/or more PCR replicates or higher sequencing depth might allow for detection of the few families and species not detected in the current study.

### Greenland halibut

Our eDNA results confirm that Greenland halibut is the most abundant species in the study area in terms of biomass, as demonstrated from trawl data for many years [48]. The offshore trawl fishery for Greenland halibut in the Davis Strait has been going on since the early 1960’s, and has grown from less than 500 tons/year in the late 1980’s to more than 7,000 tons/year, so far without any decline in stock biomass [9]. It seems fair to conclude that the fishery on the targeted species is sustainable, although the average size of individuals has declined slightly [9].

### Rockfish

Four species of rockfish (*Sebastes* spp.) are known from West Greenland waters, but the outer shelf and slope areas are heavily dominated by deepwater redfish (*Sebastes mentella*). Rockfishes were the second most abundant taxon in the trawls after Greenland halibut. The eDNA results confirmed this, as *Sebastes* spp. occurred as the third most abundant sequence in eDNA reads, just after the Greenland shark (see below). Deepwater redfish is an important commercial species in the North Atlantic including Greenland waters [49]. The West Greenland population has declined from a level of more than 1 billion individuals in 1996 to less than 100 million in 2009 [14]. In the Western Atlantic off Newfoundland northern populations are classified as "Threatened" [50].

### Greenland Shark

Our eDNA results indicate that the abundance of Greenland sharks in the study area is much higher than calculated from the trawling data, where only one shark was caught (although this specimen of 430 kg makes it one of the most abundant species in terms of total biomass). The abundance of Greenland sharks in Greenland and Arctic waters was recently considered “most likely highly abundant” or “most likely abundant” [51]. Catches were reported in 1.3% of the hauls off Southwest Greenland, but it seems that the sharks—despite slow swimming speed [52]—are able to avoid the trawls to some extent. Also supporting a high biomass, ca. 44,000 sharks were caught annually from 1890–1938 without obvious decline in catch-rates over this period [53], and catch-rates on longlines deployed on the Northeast Greenland slope are high [51]. Greenland sharks are taken as by-catch in the trawl and longline fishery, but the amount is presently unknown.

### Atlantic cod

A major shortcoming of our study is the fact that the primers used [21] do not allow for species-level taxonomic assignment of fishes in the Gadidae family. Specific primers or alternative generic primers are needed to solve this issue. Both Atlantic cod and polar cod (*Boreogadus saida*) are very common in the study area [14], and given the importance of both species in the ecosystem and economically, we consider it a high priority to develop primers that can separate these two species as well as other species in Gadidae.

### Primers in eDNA metabarcoding studies

The major inadequacy of the current eDNA metabarcoding approach is the failure of short primers to resolve some groups at the species level, which hinders sufficient biological interpretation of data. The primers used in this study perform well in resolving most taxa considering the short amplicon size [21]. However, especially for Gadidae and Sebastidae (*Sebastes* spp.), the taxonomic resolution is low, with 11 species from each group having identical sequences in our reference database. Also, for Anarhichadidae, all three *Anarhichas* species in the database have identical sequences. In all of the above instances, many of the species co-occur in the study area in SW Greenland, so the taxonomical assignment needs to be at the family or genus level (Table C in S1 File). In other cases, species with exotic distributions could be excluded. This was the case e.g. for Greenland shark (*Somniosus microcephalus*) which shared a 100% identical sequence with Pacific sleeper shark (*S*. *pacificus*), but where the latter species could be excluded due to a North Pacific distribution. Similarly, for Atlantic halibut (*Hippoglossus hippoglossus*), which shares a 100% identical sequence with Pacific halibut (*Hippoglossus stenolepis*), the latter species was excluded due to a North Pacific distribution. In these two cases, the initial assignment was thus corrected to species level (Table 1). For Clupeidae and Ammodytidae, which were only found with eDNA, identifications were upgraded to *Clupea harengus* and *Ammodytes* sp., respectively, since no other species in those families occur in Greenland (Table 1, Table C in S1 File).

### Correlation of eDNA reads with fish abundance and biomass

Perhaps *the* most apparent potential application of aquatic eDNA is the monitoring of marine fish stocks [19,20,23,25,27]. Traditional monitoring of marine fish stocks remains dependent on invasive techniques such as trawling, and the official reporting of global catches, which are essential for future estimation of sustainable fish quotas, might be off by as much as 50% [54]. Furthermore, a large number of, mainly non-commercial, fish taxa and vast areas of ocean are almost never surveyed. The current study should not be regarded as evidence that eDNA monitoring of marine fish stocks can now be initiated, but rather as a proof-of-concept that this could be future practice. This study is one of the first to demonstrate quantitative relationships between marine fish density and eDNA in a natural oceanic setting. Kelly *et al*. (2014) demonstrated in a large seawater tank that rank abundance of recovered eDNA correlated with abundance of corresponding species’ biomass [23], and Yamamoto *et al*. (2016) investigated Japanese jack mackerel (*Trachurus japonicus*) in a coastal bay area using echo sounder technology, and found a positive association between estimated eDNA concentrations and echo intensity, suggesting that eDNA concentration can reflect local biomass [26]. A relationship between eDNA and biomass has been demonstrated previously in freshwater systems for animals [18,28–30] and in terrestrial systems for plants and animals [55,56].

The prospects of this study go beyond establishing a correlation between eDNA reads and biomass for fish assemblages. Interpolated eDNA read abundance data showed good overlap with catch data for highly abundant commercial species (Greenland halibut and rockfish) (Fig 5). Such data can be incorporated into more elaborate models with long-term data to test estimation of fish stocks in more detail, or to monitor the continuous northward dispersal of a marine taxa due to global change [1,2].

### Influences from other factors

It is obvious that an initial relationship between fish density and eDNA sequence abundance can be obscured by several sources of error. The final number of eDNA sequences recovered for each taxon is a representation of the unknown original number of eDNA sequences in the water, which is again only (supposedly) a representation of the actual density (individuals and biomass) of that taxon [57]. The relationship between fish density and eDNA abundance depends on e.g. taxon- and age-specific shedding rates, specific eDNA degradation rates in the given environment, and non-local eDNA transported with sea currents. Furthermore, biotic and abiotic factors such as microbial activity, temperature, salinity and pH can influence eDNA survival and availability [58,59]. Assuming that the eDNA in an area is a reasonable representation of the fish density in the same area, this relationship is not necessarily retained in the final eDNA data [57]. The relation between true on-site eDNA abundance and final eDNA read abundance will be influenced by i) the water volume sampled and area covered, ii) the amount of eDNA captured on the filter and the proportion of this eDNA subsequently obtained from DNA extraction, iii) the primer affinity for each taxon present, iv) stochasticity in PCR and/or sequencing, v) errors generated during PCR and/or sequencing [60], and vi) bioinformatic data cleaning and assignment of sequence reads to taxa, and as a significant part of the latter, the taxonomic coverage of the reference database. Minimizing false negatives and false positives in eDNA results will be essential for making defensible ecological inferences [61,62]. The former depends on increasing sampling intensity, PCR replicates and sequencing depth [21,63], while the latter depends on serious precautions taken to avoid field- and laboratory-based contamination as well as bioinformatic cleaning of PCR and sequencing errors [38,60,62]. In this study, only usual lab contaminants as well as a few more serious point contaminations with fish was observed. Only more stringent sampling and lab practices can exclude these incidences in eDNA studies, but it is unlikely that studies using high-throughput sequencing will be completely free of spurious reads. This underpins the importance of taxonomic expertise and careful consideration when analyzing and presenting eDNA results.

For marine eDNA, oceanic currents are probably one of the greatest influences to take into account if inferences are to be made on the proximity of target organisms to their eDNA traces. Transport of eDNA within ecosystems remains a challenge in flowing waters [29,64–66] and marine environments [19,24], where long-distance transport is possible, even from terrestrial sources [67]. Several studies, however, have documented rapid degradation of aquatic eDNA fragments beyond detectability within days to weeks [18,19,31,32,59], and Foote *et al*. (2012) showed that an eDNA signal from harbour porpoise (*Phocoena phocoena*) was evident only within short distances (metres) of animals [22]. These results suggest that at least the strongest eDNA signals come from individuals that were in close proximity to where the water sample was taken. Nevertheless, only one study directly investigates degradation rates in seawater [19] and influences from sea currents remain to be studied. Hence, degradation rates as well as eDNA dispersal through sea currents should be a high priority in future research on seawater eDNA. The hydrographic conditions in the study area are dominated by the West Greenland current (WGC) that runs northward along the west coast of Greenland. The WGC is a mixture of cold water from the East Greenland Current, which is found near the coast of SW Greenland, and of water from the warmer Irminger current—a branch of the Golf Stream [68], which is found at greater depths and further off shore. The two currents gradually mix as the WGC runs northward. The hydrographic conditions in the study area are mainly influenced by Irminger water coming from the south. This is in concordance with our eDNA results, where we do not detect any truly arctic fish species such as *Arctogadus glacialis*, which is found north of the sampling site.

The above influences may also partly explain the incongruence between the two methods for some fish families (Figs 2 and 4A). Obviously, the two methods are different in what species they primarily target. As mentioned above, some of the most apparent incongruences may be explained by the taxon being very rare and therefore easily missed by eDNA or trawling (Platytroctidae, Serrivomeridae, Oneirodidae etc.), by the ability of the taxon to avoid trawls (Somniosidae), or by the taxon being pelagic (Clupeidae, Paralepididae) and therefore not efficiently targeted with bottom trawling. Nonetheless, there was good overall resemblance between eDNA and trawling results when comparing the total number of sites that a family was detected in (Fig 3). This is important for making qualitative assessments of marine biota using water samples. However, a larger number of samples are needed to test this further.

### Future directions

“*Catchability*” is a central concept in fish stock assessments [69], and refers to the relationship between the true abundance of the fish resource and the efficiency of the fishing method (e.g. trawling). In other words, the catch is not necessarily representative of the true abundance and biomass of fish present in a surveyed area of the ocean [70,71]. This is central to the results obtained using eDNA approaches, since a perfect correlation between eDNA read abundance and fish catch cannot necessarily be expected. It is well known that using several different monitoring methods simultaneously yields an overall better qualitative estimation of marine fish faunas [16], and at a coastal site the eDNA approach has been shown to outperform each of nine traditional monitoring methods with respect to the number of fish species detected [19,21]. Making quantitative inferences from eDNA represents a bigger challenge since more factors influence both the catch using traditional monitoring methods as well as the amount of recovered eDNA sequence reads, as described above. Hence, if eDNA is to become a supplementary or alternative approach in fish stock assessment and monitoring of marine resources in general, the challenges described above need to be further investigated. Particularly, the relationship between the abundance of fish in an area and the corresponding eDNA in the surrounding water, at different spatial and temporal scales [26,57]. An obvious next step would be comparative stratified random bottom trawl surveys in parallel with dense water samplings analyzed with deep high-throughput sequencing of minimum 12 PCR replicates per sample [21,63], in order to exhaustively detect rare taxa. Direct sequencing techniques independent of initial taxon-specific PCR to avoid amplification bias also represent an appealing approach.

## Conclusion

We here present results that suggest a correspondence between fish density (abundance and biomass) and marine eDNA sequence reads produced from Illumina high-throughput sequencing. These are some of the first proof-of-concept studies that show a possibility for marine fish stock assessment using water samples. We stress that a direct application of this would still require that several factors are more rigorously investigated. However, applying eDNA to stock assessment could potentially be very fruitful not only for biodiversity surveys of marine fishes and other organisms, but also for science, society and the global economy.

## Supporting Information

S1 File This file contains all Supporting Figures (A-E) and Tables (A-C). **Figure A in S1 File**. Histograms of input data before (above) and after (below) log transformation, showing left skewed data and approximation to normal distribution after transformation. Figure shows data for eDNA, biomass and number of individuals, respectively. **Figure B in S1 File**. Relationship between eDNA reads and biomass for each sample individually for all families. One sample (davis14) has been removed since there are no corresponding points. This figure corresponds to Fig 6, but partitioned into samples. **Figure C in S1 File**. Relationship between eDNA reads and number of individuals for each sample individually for all families. One sample (davis14) has been removed since there are no corresponding points. This figure corresponds to Fig 6, but partitioned into samples. **Figure D in S1 File**. Relationship between eDNA read frequencies and biomass (A) as well as number of individuals (B) at lowest possible taxonomic level. Results are shown for all fish taxa detected using both methods across all samples. **Figure E in S1 File**. Correlation of eDNA read abundance and tissue derived DNA concentrations added to the mock sample. **Table A in S1 File**. Information on sampling sites for this study. Table shows sample names, geographical position and depths. **Table B in S1 File**. Mitochondrial 12S rRNA gene (partial) target sequences generated from tissue samples of Greenland fish species for the local reference database of the current study. **Table C in S1 File.** Greenlandic fish species. Table shows all fish species recorded in Greenland within the 31 families found in this study, along with information on general abundance (expectance of finding the species) (based on Møller *et al*. 2010, [10]) and coverage in local 12S rRNA gene database. This information is used for adjusting the taxonomic assignments given to eDNA reads (Table 1).
(PDF)
