# Supplementary material for: Environmental DNA from Seawater Samples Correlate with Trawl Catches of Subarctic, Deepwater Fishes
Source: PLoS One. 2016 Nov 16;11(11):e0165252. doi: 10.1371/journal.pone.0165252 (PMC5112899; doi:10.1371/journal.pone.0165252)
Supplement: S1 File — Figure A in S1 File. Histograms of input data before (above) and after (below) log transformation, showing left skewed data and approximation to normal distribution after transformation. Figure shows data for eDNA, biomass and number of individuals, respectively. Figure B in S1 File. Relationship between eDNA reads and biomass for each sample individually for all families. One sample (davis14) has been removed since there are no corresponding points. This figure corresponds to Fig 6, but partitioned into samples. Figure C in S1 File. Relationship between eDNA reads and number of individuals for each sample individually for all families. One sample (davis14) has been removed since there are no corresponding points. This figure corresponds to Fig 6, but partitioned into samples. Figure D in S1 File. Relationship between eDNA read frequencies and biomass (A) as well as number of individuals (B) at lowest possible taxonomic level. Results are shown for all fish taxa detected using both methods across all samples. Figure E in S1 File. Correlation of eDNA read abundance and tissue derived DNA concentrations added to the mock sample. Table A in S1 File. Information on sampling sites for this study. Table shows sample names, geographical position and depths. Table B in S1 File. Mitochondrial 12S rRNA gene (partial) target sequences generated from tissue samples of Greenland fish species for the local reference database of the current study. Table C in S1 File. Greenlandic fish species. Table shows all fish species recorded in Greenland within the 31 families found in this study, along with information on general abundance (expectance of finding the species) (based on Møller et al. 2010, [10]) and coverage in local 12S rRNA gene database. This information is used for adjusting the taxonomic assignments given to eDNA reads (Table 1). (PDF) [file pone.0165252.s001.pdf]

Supporting Information for:

Environmental DNA from seawater samples correlate with trawl catches of subarctic, deepwater fishes

Philip Francis Thomsen[1*], Peter Rask Møller[2], Eva Egelyng Sigsgaard[2], Steen Wilhelm Knudsen[2], Ole Ankjær Jørgensen[3,4], Eske Willerslev[1,5,6*]

[1] Centre for GeoGenetics, Natural History Museum of Denmark, University of Copenhagen, Denmark.

[2] Section for EvoGenomics, Natural History Museum of Denmark, University of Copenhagen, Denmark.

[3] Technical University of Denmark, National Institute of Aquatic Resources, Charlottenlund, Denmark.

[4] Greenland Institute of Natural Resources, Nuuk, Greenland.

[5] Department of Zoology, University of Cambridge, Cambridge, UK.

[6] Wellcome Trust Sanger Institute, Wellcome Genome Campus, Hinxton, Cambridge, UK.

*) E-mail: pfthomsen@snm.ku.dk (PFT); ewillerslev@snm.ku.dk (EW)

Figure A. Histograms of input data before (above) and after (below) log transformation, showing left skewed data and approximation to normal distribution after transformation. Figure shows data for eDNA, biomass and number of individuals, respectively.

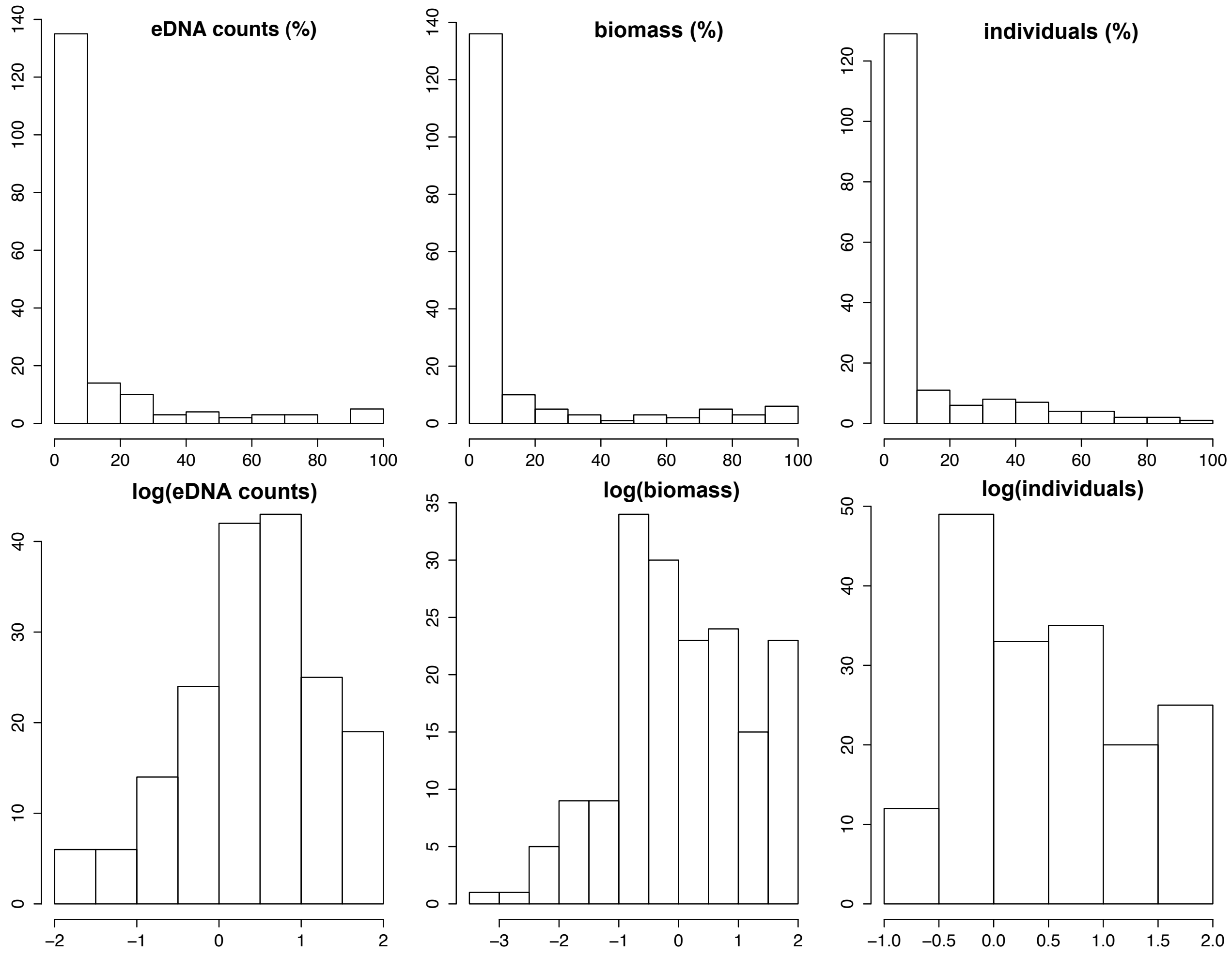

Figure B. Relationship between eDNA reads and biomass for each sample individually for all families. One sample (Davis_14) has been removed since there are no corresponding points. This figure corresponds to Fig 6, but partitioned into samples.

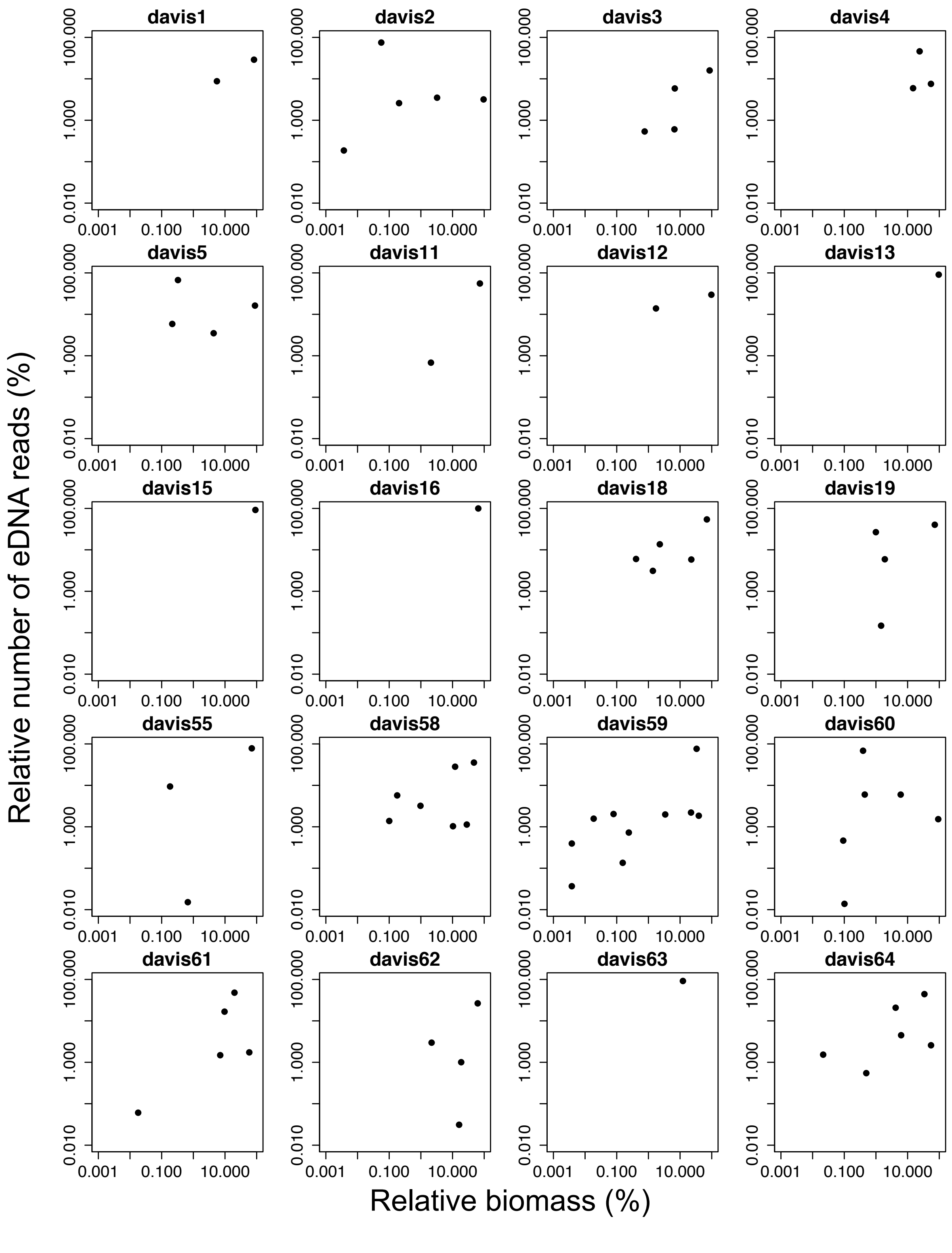

Figure C. Relationship between eDNA reads and number of individuals for each sample individually for all families. One sample (Davis_14) has been removed since there are no corresponding points. This figure corresponds to Fig 6, but partitioned into samples.

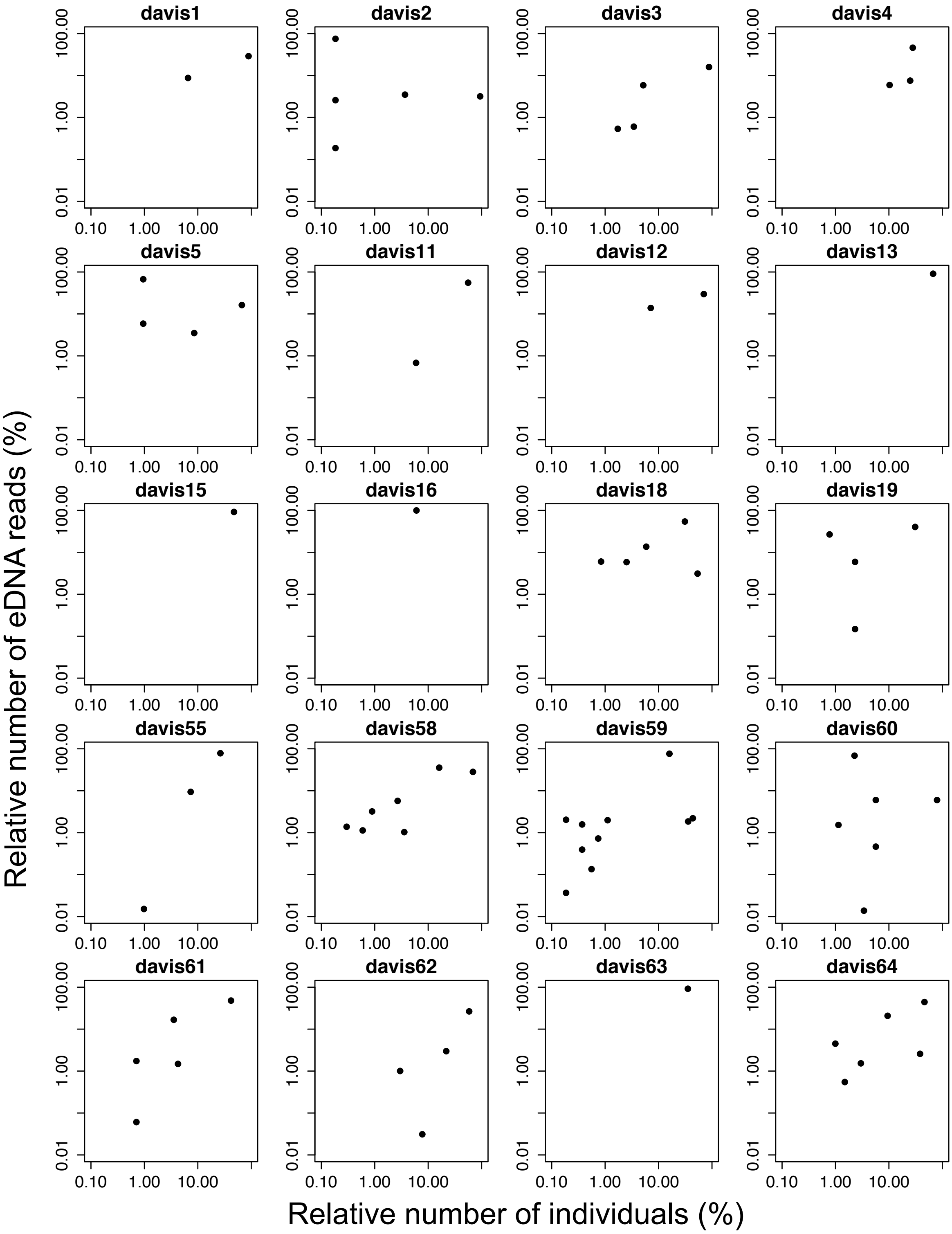

Figure D. Relationship between eDNA read frequencies and biomass (A) as well as number of individuals (B) at lowest possible taxonomic level. Results are shown for all fish taxa detected using both methods across all samples.

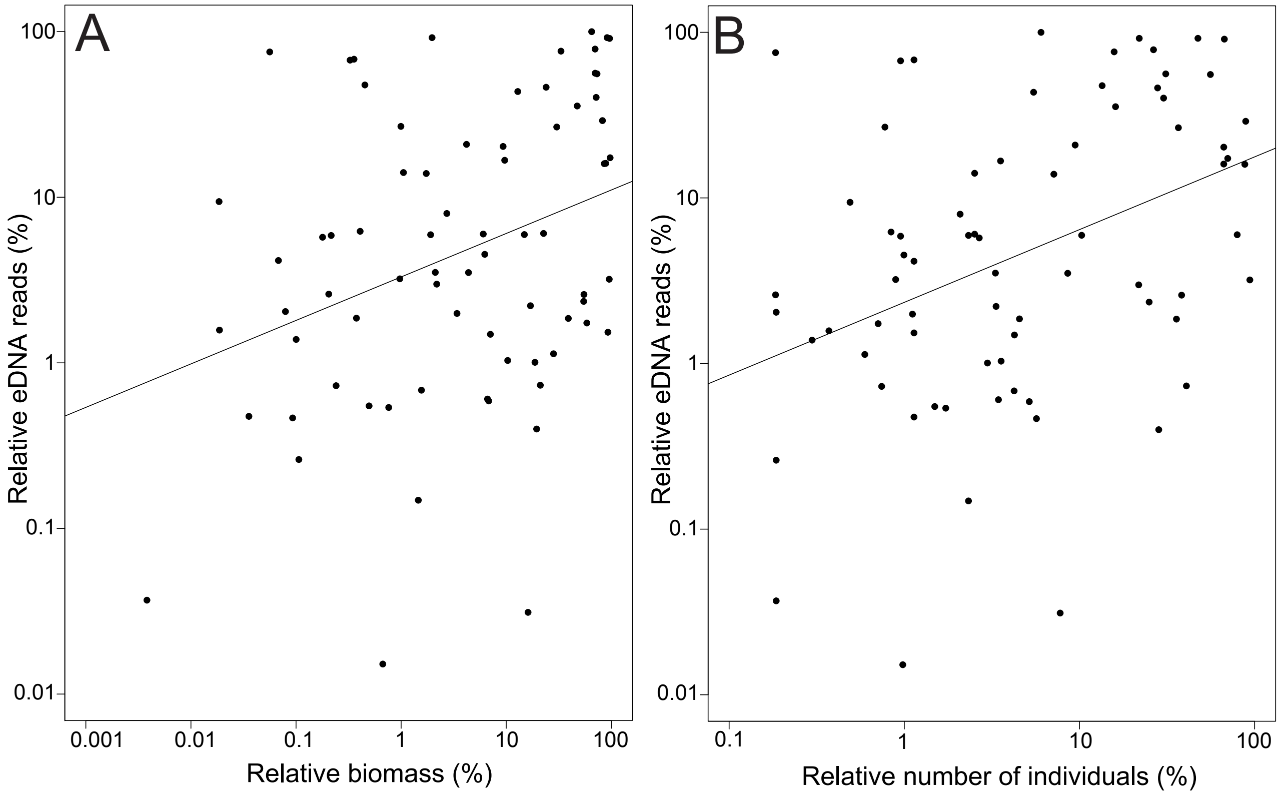

Figure E. Correlation of eDNA read abundance and tissue derived DNA concentrations added to the mock sample.

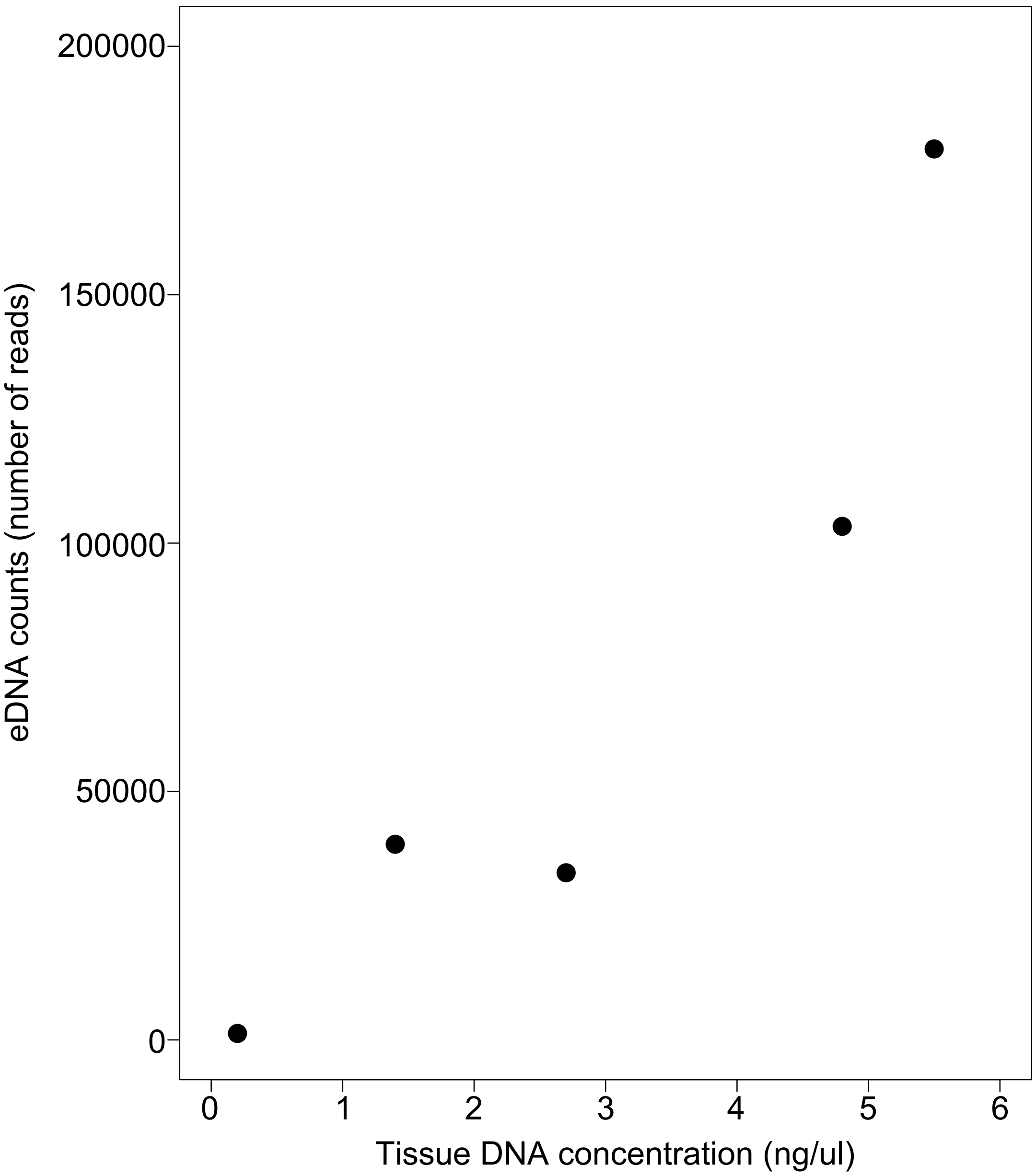

Table A. Information on sampling sites for this study. Table shows sample names, geographical position and depths.

Sample	Latitude, N	Longitude, W	Depth (m)
Davis_1	65.2199	-55.0316	585
Davis_2	65.4722	-55.3298	571.5
Davis_3	65.7282	-56.011	628
Davis_4	65.498	-56.7579	672.5
Davis_5	65.2439	-56.0266	781.5
Davis_11	64.6832	-55.7001	951
Davis_12	64.7406	-56.2167	858
Davis_13	64.7218	-55.0436	917.5
Davis_14	64.753	-57.2995	801.5
Davis_15	64.4584	-57.715	846.5
Davis_16	64.403	-57.4151	842
Davis_18	64.4731	-57.0501	826
Davis_19	64.3894	-56.9829	792
Davis_55	64.2599	-54.5698	822
Davis_58	64.1498	-54.1375	842.5
Davis_59	64.1302	-54.0197	735
Davis_60	64.2237	-54.0267	495.5
Davis_61	63.7558	-52.3561	206
Davis_62	63.7273	-52.3369	259.5
Davis_63	63.6445	-52.3538	188
Davis_64	63.4969	-52.3301	466.5

Table B. Mitochondrial 12S rRNA gene (partial) target sequences generated from tissue samples of Greenland fish species for the local reference database of the current study.

Species	Specimen voucher	Sequence (5'-'3)
Alepocephalus agassizii	ZMUC P2394941	CCCCGAACCCCAACCTTTAAAAGTAAATAACAAAATTAACTCACCAAGGGGAGGCAAGTCGTAA
Anarhichas denticulatus	ZMUC P2394942	CCCCAAGCCCACCAACCTAATTAACTAAACCCTAATAATCGCAAAGGGGAGGCAAGTCGTAA
Anarhichas minor	ZMUC P75870	CCCCAAGCCCACCAACCTAATTAACTAAACCCTAATAATCGCAAAGGGGAGGCAAGTCGTAA
Antimora rostrata	ZMUC P375462	CTCCAAATAAAACCTAAGAGTTACCTAAAACATTTTATTAATTCAAGGAGAGGCAAGTCGTAA
Arctozenus risso	ZMUC P2394943	CCCCAAGTCTAGTCCAACAAGTCCATAAAACAACTTTCTTATAAAGGGGAGGCAAGTCGTAA
Bathylagus euryops	ZMUC P191154	CCCCAAGTCCACTCTCATAGATAATTAAGAAATCAACCGGACTAAGGGGAGGCAAGTCGTAA
Bathyraja spinicauda	ZMUC P2394944	CCTCAATTTCAATCTTTTCCTTTTATAAATAGTCTTGTTTACAAGAGGAGGCAAGTCGTAA
Benthosema glaciale	ZMUC P2394945	CCCCAAACCCAACCAACCCCTCACTAACATATATCACCCCCTGGATAAAGGGGAGGCAAGTCGTAA
Boreogadus saida	ZMUC P375405	CTCCAAATAAACCCTAGATATTACCTAAAATGCTTTTTATAATAAGGGGAGGCAAGTCGTAA
Borostomias antarcticus	ZMUC P2394946	CCCCAAACCCACACCTTCCAGTAACTAAAAAGACAACCGGACCAAGGGGAGGCAAGTCGTAA
Cauliodus sloani	ZMUC P2394947	CCGCTAGTTCCCGCATCTCTTGTACCAACAAAATAGCAAGACAAAGCAGAGGCAAGTCGTAA
Centroscyllium fabricii	ZMUC P07197	CCTCAACAAAACCTTTATTTTTTTTACATAAAACCTTTATTTTATAAAGAGGAGGCAAGTCGTAA
Coryphaenoides guentheri	ZMUC P2394948	CTCCGAGATATCTTTATTAATGCCTAATACGTAATAATTCAAGAAGGCGAGAAAAGTCGTAA
Coryphaenoides rupestris	ZMUC P375899	CTCCAAAACAAATTCCTTTGTACGTAACACATAAAATAAAACAAGGCGAGATAAGTCGTAA
Cottunculus thomsonii	ZMUC P814846	CCCCAAAAGCCCCAGCCAGTTAATTAAAACCTAATAATCAAAGAGGGGAGGCAAGTCGTAA
Cyclopterus lumpus	ZMUC P821740	CCCCAAAACCACACCCCCGTTAATTAAAACCTAATAACCAACAAGGGGAGGCAAGTCGTAA
Cyclothone microdon	ZMUC P2014787	CCCCAAAATCTCTCTCATAATTCCTAAAACCGGGAACTTCAGAAGGGGAGGCAAGTCGTAA
Gaidropsarus argentatus	ZMUC P2394949	CTCCAAATACTCATAATTATTTACCTAAAATATTTAAACTAACAAGGAGAGGCAAGTCGTAA
Gaidropsarus ensis	ZMUC P375255	CTCCAAATACTCATAATTATTTACCTAAAATATTTAAACTAACAAGGAGAGGCAAGTCGTAA
Hippoglossoides platessoides	ZMUC P855805	CCCCAAGCATCCTGAATAAACCTAATTAAAAGCCAACAGACCGCGAAGGGGAGGAAAGTCGTAA
Holtbyrnia anomala	ZMUC P2394950	CCCCGCACCCCCAACCTAAAGTAAATAACAAAATTAACTCACCAAGGGGAGGCAAGTCGTAA
Lampanyctus intricatus	ZMUC P2393471	CCCCAAGTCCTACCCTACCATAACCTATAGCCTTATATGGATAAAGGGGAGGCAAGTCGTAA
Lampanyctus crocodilus	ZMUC P2394951	CCCCAAGTCCCACCCTACCATAACCTATAGCCTTATATGGATAAAGGGGAGGCAAGTCGTAA
Lepidion eques	ZMUC P375406	CTCCAAATAAAACCTAAAAGTTACCTAAAACATTTTATTCACTCAAGGAGAGGCAAGTCGTAA
Macrourus berglax	ZMUC P375333	CCCCGAAACAACCTCTTTATTCCTAATAATTTAAATAAAACAAGGTGAGATAAGTCGTAA
Magnisudis atlantica	ZMUC P2393498	CCCCAAATATAACCCACCTAGTTAATAAAAAACCTTTTAAACGAAGGGGAGGCAAGTCGTAA
Malacosteus niger	ZMUC P2394952	CCCCGAGTTCATCGTGCCCGCTAATTAAGAAGTGAGTCAAACAAAGGGGAGGCAAGTCGTAA
Mallotus villosus	ZMUC P191156	CTCCAAGTTCACTAAATTTGGTTCTTAACAAGATCACCGAACAAAGGGGAGGCAAGTCGTAA
Melanogrammus aeglefinus	ZMUC P375933	CTCCAAATAAACCCTAGATATTACCTAAAATGCTTTTTATAATAAGGGGAGGCAAGTCGTAA
Molva dypterygia	ZMUC P2394846	CTCCAAATAAACCCTGAATATTACCTAAAATGTTTTATATAATAAGGGGAGGCAAGTCGTAA
Myctophum punctatum	ZMUC P2393486	CCCCGAAGTCTCTCTACAGCCTTAACTTATAGCACAAAACTGACAAAGAGGAGGAAAGTCGTAA
Nezumia bairdii	ZMUC P2394953	CTCCGAGATATCTTTATTAATGCCTAATACGTAATAATTCAAGAAGGCGAGAAAAGTCGTAA
Paraliparis copei	ZMUC P821757	CTCCAAAATTAACGCCAAATTAACTAAAACCTAATTAAACAAAAAGGAGAGGCAAGTCGTAA
Rajella bigelowi	ZMUC P08737	CCTCAACCTATGCTACTCTATTCTATAAATAAATTTTTATCACAAGAGGAGGCAAGTCGTAA
Rajella fyllae	ZMUC P08634	CCTCAACCTATGTTACTCTATTTTATAAATAAATTTTTATCACAAGAGGAGGCAAGTCGTAA
Amblyaja radiata	ZMUC P08636	CCTCAACCTAAGCTACTCTATTTTATAAATAAATTTTTATCACAAGAGGAGGCAAGTCGTAA
Reinhardtius hippoglossoides	ZMUC P855804	CCCCAAGCCCCCTGAACTAAACTAATTAAAACCCAACAACCCGCGAAGGGGAGGAAAGTCGTAA
Scopelosaurus lepidus	ZMUC P2394954	CCCCCAGCACCCGGCCTGACCGTTATTAAACTGATTATGTAACAAGGGGAGGCAAGTCGTAA
Sebastes mentella	ZMUC P791418	CCCCAAGCCTATCACTTTAAATAATTAAAAACCCAAAAATCGCGGAGGGGAGGCAAGTCGTAA
Sebastes norvegicus	ZMUC P2394847	CCCCAAGCCTATCACTTTAAATAATTAAAAACCCAAAAATCGCGGAGGGGAGGCAAGTCGTAA
Sigmops bathyphilus	ZMUC P2394955	CCCCGAGCCCACCAACACATCACTAAAAATCAACTAACTAAGGGGAGGCAAGTCGTAA
Somniosus microcephalus	ZMUC P2394956	CCTCAACAAAACCTTTACTTTTTATAAAAAAATTATTTTACTAAGAGGAGGCAAGTCGTAA
Stomias boa	ZMUC P2014774	CCCCAAGTTCACCTCTATAAGTAAGTAAAAGAACTATAGAACTAAGGGGAGGCAAGTCGTAA
Synaphobranchus kaupii	ZMUC P321354	CCTCGAAATAAATTCAAAACATATATAAACACTAAAATACAAGAAAGAGGAGACAAGTCGTAA
Trachyrincus murrayi	ZMUC P375245	CTCCGAATGAACCCTTGATGATACCTAATATGTTTTACCAATTAAGGAGAGGCAAGTCGTAA

Table C. Greenlandic fish species. Table shows all fish species recorded in Greenland within the 31 families found in this study, along with information on general abundance (expectance of finding the species) (based on Møller *et al*. 2010, [10]) and coverage in local 12S rRNA gene database. This information is used for adjusting the taxonomic assignments given to eDNA reads (Table 1).

Family	Species	Local database	Commonness
Alepocephalidae	*Alepocephalus agassizii* Goode & Bean, 1883	x	common
Alepocephalidae	*Alepocephalus bairdii* Goode & Bean, 1879	x	common
Alepocephalidae	*Bajacalifornia megalops* (Lütken, 1898)	x	rather common
Alepocephalidae	*Bathylaco nigricans* Goode & Bean, 1896	x	very rare
Alepocephalidae	*Bathytroctes microlepis* Günther, 1878	x	very rare
Alepocephalidae	*Einara edentula* (Alcock, 1892)	-	very rare
Alepocephalidae	*Photostylus pycnopterus* Beebe, 1933	-	very rare
Alepocephalidae	*Rouleina attrita* (Vaillant, 1888)	-	common
Alepocephalidae	*Rouleina maderensis* Maul, 1948	-	common
Alepocephalidae	*Xenedermichthys copei* (Gill, 1884)	x	common
Ammodytidae	*Ammodytes dubius* Reinhardt, 1837	x	common
Ammodytidae	*Ammodytes marinus* Raitt, 1934	x	common
Anarhichadidae	*Anarhichas denticulatus* Krøyer, 1845	x	very common
Anarhichadidae	*Anarhichas lupus* Linnaeus, 1758	x	very common
Anarhichadidae	*Anarhichas minor* Olafsen & Poulsen, 1772	x	very common
Argentinidae	*Argentina silus* (Ascanius, 1775)	x	common
Arhynchobatidae	*Bathyraja spinicauda* (Jensen, 1914)	x	rather common
Bathylagidae	*Bathylagus euryops* Goode & Bean, 1896	x	common
Bathylagidae	*Melanolagus bericoides* (Borodin, 1929)	-	rare
Clupeidae	*Clupea harengus* Linnaeus, 1758	x	relatively rare
Cyclopteridae	*Cyclopterus lumpus* Linnaeus, 1758	x	common
Cyclopteridae	*Cyclopteropsis mcalpini* (Fowler, 1914)	-	very rare
Cyclopteridae	*Eumicrotremus derjugini* Popov, 1926	-	very rare
Cyclopteridae	*Eumicrotremus spinosus* (Fabricius, 1776)	-	very common
Etmopteridae	*Centroscyllium fabricii* (Reinhardt, 1825)	x	common
Etmopteridae	*Etmopterus princeps* Collett, 1904	x	very rare
Gadidae	*Arctogadus glacialis* (Peters, 1874)	x	common
Gadidae	*Boreogadus saida* (Lepechin, 1774)	x	very common
Gadidae	*Gadus macrocephalus* Tilesius, 1810	x	very common
Gadidae	*Gadus morhua* Linnaeus, 1758	x	very common
Gadidae	*Melanogrammus aeglefinus* (Linnaeus, 1758)	x	rare
Gadidae	*Merlangius merlangus* (Linnaeus, 1758)	x	rare
Gadidae	*Micromesistius poutassou* (Risso, 1826)	x	rare

Gadidae	*Pollachius virens* (Linnaeus, 1758)	x	rare
Gadidae	*Trisopterus esmarkii* (Nilsson, 1855)	x	rare
Gaidropsaridae	*Gaidropsaurus argentatus* (Reinhardt, 1837)	x	common
Gaidropsaridae	*Gaidropsaurus ensis* (Reinhardt, 1837)	x	common
Gonostomatidae	*Cyclothone braueri* Jespersen & Tåning, 1926	-	rather rare
Gonostomatidae	*Cyclothone microdon* (Günther, 1878)	x	common
Gonostomatidae	*Sigmops bathyphilus* (Vaillant, 1888)	x	rather common
Gonostomatidae	*Sigmops elongatus* (Günther, 1878)	-	very rare
Liparidae	*Careproctus micropus* (Günther, 1887)	-	very rare
Liparidae	*Careproctus reinhardti* (Krøyer, 1862)	-	very common
Liparidae	*Careproctus kidoi* Knudsen & Møller, 2008	-	rare
Liparidae	*Liparis fabricii* Krøyer, 1847	-	very common
Liparidae	*Liparis gibbus* Bean, 1881	-	common
Liparidae	*Liparis tunicatus* Reinhardt, 1837	-	common
Liparidae	*Paraliparis bathybius* (Collett, 1879)	-	common
Liparidae	*Paraliparis copei* Goode & Bean, 1896	x	common
Liparidae	*Paraliparis garmani* Burke, 1912	-	common
Liparidae	*Psednos christinae* Andriashev, 1992	-	very rare
Liparidae	*Psednos gelatinosus* Chernova, 2001	-	very rare
Liparidae	*Psednos groenlandicus* Chernova, 2001	-	very rare
Liparidae	*Psednos melanocephalus* Chernova & Stein, 2002	-	very rare
Liparidae	*Psednos micruroides* Chernova, 2001	-	very rare
Liparidae	*Rhodichthys regina* Collett, 1879	-	common
Lotidae	*Brosme brosme* (Ascanius, 1772)	-	rare
Lotidae	*Molva dypterygia* (Pennant, 1784)	x	rare
Lotidae	*Molva molva* (Linnaeus, 1758)	x	very rare
Macrouridae	*Coryphaenoides armatus* Hector, 1875	-	very rare
Macrouridae	*Coryphaenoides brevibarbis* (Goode & Bean, 1896)	-	common
Macrouridae	*Coryphaenoides carapinus* Goode & Bean, 1883	-	very rare
Macrouridae	*Coryphaenoides guentheri* (Vaillant, 1888)	x	common
Macrouridae	*Coryphaenoides longifilis* Günther, 1877	-	very rare
Macrouridae	*Coryphaenoides mediterranea* (Giglioli, 1893)	-	very rare
Macrouridae	*Coryphaenoides rupestris* Gunnerus, 1765	x	very common
Macrouridae	*Macrourus berglax* Lapépede, 1801	x	very common
Macrouridae	*Nezumia aequalis* (Günther, 1878)	-	very rare
Macrouridae	*Nezumia bairdii* (Goode & Bean, 1877)	x	common
Macrouridae	*Trachyrincus murrayi* Günther, 1887	x	common
Moridae	*Antimora rostrata* (Günter, 1878)	x	very common
Moridae	*Guttigadus latifrons* (Holt & Byrne, 1908)	-	very rare
Moridae	*Halargyreus johnsonii* Günther, 1862	x	very rare

Moridae	*Lepidion eques* (Günther, 1887)	x	very rare
Myctophidae	*Benthosema glaciale* (Reinhardt, 1837)	x	very common
Myctophidae	*Lampadena speculigera* Goode & Bean, 1896	-	common
Myctophidae	*Lampanyctus crocodilus* (Risso, 1810)	X	rare
Myctophidae	*Lampanyctus intricarius* Tåning, 1928	X	rare
Myctophidae	*Lampanyctus macdonaldi* (Goode & Bean, 1896)	X	very common
Myctophidae	*Myctophum punctatum* Rafinesque, 1810	x	very common
Myctophidae	*Notoscopelus kroeyeri* (Malm, 1861)	x	very common
Myctophidae	*Protomyctophum arcticum* (Lütken, 1892)	x	very common
Notacanthidae	*Notacanthus chemnitzii* Block, 1788	x	common
Notacanthidae	*Polyacanthonotus rissoanus* (De Filippi & Verany, 1859)	x	common
Notosudidae	*Scopelosaurus lepidus* (Krefft & Maul, 1955)	-	common
Oneirodidae	*Chaenophryne draco* Beebe, 1932	-	very rare
Oneirodidae	*Chaenophryne longiceps* Regan, 1925	-	very rare
Oneirodidae	*Danaphryne nigrifilis* (Regan & Trewavas, 1932)	-	very rare
Oneirodidae	*Dolopichthys longicornis* Parr, 1927	-	very rare
Oneirodidae	*Lophodolos acanthognathus* Regan, 1925	-	very rare
Oneirodidae	*Oneirodes eschrichtii* Lütken, 1871	-	rare
Oneirodidae	*Oneirodes macrosteus* Pietsch, 1974	-	very rare
Oneirodidae	*Phyllorhinichthys balushkini* Pietsch, 2004	-	very rare
Oneirodidae	*Phyllorhinichthys micractis* Pietsch, 1969	-	very rare
Oneirodidae	*Spiniphryne gladisfenae* (Beebe, 1932)	-	very rare
Osmeridae	*Mallotus villosus* (Müller, 1776)	x	very common
Paralepididae	*Arctozenus risso* (Bonaparte, 1840)	x	very common
Paralepididae	*Magnisudis atlantica* (Krøyer, 1868)	x	relatively rare
Paralepididae	*Paralepis coregonoides* Risso, 1820	-	very common
Platytroctidae	*Barbantus curvifrons* (Roule and Angel, 1931)	-	very rare
Platytroctidae	*Holtbyrnia anomala* Krefft, 1980	x	common
Platytroctidae	*Holtbyrnia macrops* Maul, 1957	-	common
Platytroctidae	*Maulisia mauli* Parr, 1960	x	rather rare
Platytroctidae	*Maulisia microlepis* Sazonov & Golovan, 1976	-	rather common
Platytroctidae	*Normichthys operosus* Parr, 1951	x	rather rare
Platytroctidae	*Platytroctes apus* Günther, 1878	x	very rare
Platytroctidae	*Sagamichthys schnakenbecki* (Krefft, 1953)	-	very rare
Platytroctidae	*Searsia koefoedi* Parr, 1937	-	rather common
Pleuronectidae	*Glyptocephalus cynoglossus* (Linnaeus, 1758)	-	rather common
Pleuronectidae	*Hippoglosoides platessoides* (Fabricius, 1780)	x	very common
Pleuronectidae	*Hippoglossus hippoglossus* (Linnaeus, 1758)	x	common
Pleuronectidae	*Microstomus kitt* (Walbaum, 1792)	x	rare
Pleuronectidae	*Pleuronectes platessa* Linnaeus, 1758	x	rare

Pleuronectidae	*Reinhardtius hippoglossoides* (Walbaum, 1792)	x	very common
Psychrolutidae	*Cottunculus microps* Collett, 1875	-	very rare
Psychrolutidae	*Cottunculus thomsonii* (Günther, 1882)	x	common
Psychrolutidae	*Psychrolutes subspinosus* (Jensen, 1902)	-	rare
Rajidae	*Amblyraja hyperborea* (Collett, 1879)	x	very common
Rajidae	*Amblyraja jenseni* (Bigelow & Schroeder, 1950)	-	very rare
Rajidae	*Amblyraja radiata* (Donovan, 1808)	x	very common
Rajidae	*Malacoraja spinacidermis* (Barnard, 1923)	-	rare
Rajidae	*Rajella bathyphila* (Holt & Byrne, 1908)	-	rather rare
Rajidae	*Rajella bigelowi* (Stehmann, 1978)	x	rather rare
Rajidae	*Rajella fyllae* (Lütken, 1888)	x	common
Rajidae	*Rajella lintea* (Fries, 1838)	-	very rare
Sebastidae	*Helicolenus dactylopterus* (Delaroche, 1809)	x	very rare
Sebastidae	*Sebastes fasciatus* Storer, 1856	-	very rare
Sebastidae	*Sebastes norvegicus* (Ascanius, 1772)	x	very common
Sebastidae	*Sebastes mentella* Travin, 1951	x	very common
Sebastidae	*Sebastes viviparus* Krøyer, 1845	-	very rare
Serrivomeridae	*Serrivomer beanii* Gill & Ryder, 1883	x	common
Somniosidae	*Centroscymnus coelolepis* Barbosa du Bocage & de Brito Capello, 1864	-	very rare
Somniosidae	*Somniosus microcephalus* (Bloch & Schneider, 1801)	x	common
Stomiidae	*Borostomias antarcticus* (Lönnberg, 1905)	x	common
Stomiidae	*Chauliodus sloani* Bloch & Schneider, 1801	x	common
Stomiidae	*Malacosteus niger* Ayres, 1848	x	rather common
Stomiidae	*Rhadinesthes decimus* (Zugmayer, 1911)	-	rare
Stomiidae	*Stomias boa* Risso, 1810	x	common
Stomiidae	*Trigonolampa miriceps* Regan & Trewavas, 1930	-	very rare
Synaphobranchidae	*Histiobranchus bathybius* (Günther, 1877)	-	very rare
Synaphobranchidae	*Synaphobranchus kaupii* Johnson, 1862	x	very common
